# Eye-of-the-tiger Sign in Neurodegeneration with Brain Iron Accumulation

**DOI:** 10.7759/cureus.4936

**Published:** 2019-06-18

**Authors:** Madeline Goldberg, Khizar Malik, Rekha Jiswant, Aunali S Khaku

**Affiliations:** 1 Internal Medicine, University of Central Florida College of Medicine, Orlando, USA; 2 Neurology, University of Central Florida College of Medicine, Orlando, USA

**Keywords:** mri, nbia, hallervorden-spatz, eye-of-the-tiger-sign

## Abstract

A 68-year-old male patient presented to the neurology clinic with tremor, lightheadedness, and a history of syncope. Exam showed mild Parkinsonism. Neuroimaging revealed symmetric lesions of the globus pallidus (the eye-of-the-tiger sign) concerning for neurodegeneration with brain iron accumulation (NBIA). Genetic panel for NBIA was ordered, specifically pantothenate kinase-associated neurodegeneration (PKAN), including pantothenate kinase 2 (PanK2) - the genetic marker for the pantothenate kinase enzyme.

## Introduction

Neurodegeneration with brain iron accumulation (NBIA) presents with characteristic MRI abnormalities in the basal ganglia [1]. This case report presents a 68-year-old male suffering from Parkinsonism (tremor/gait abnormalities) and syncope with MRI fluid-attenuated inversion recovery (FLAIR) showing an eye-of-the-tiger sign. A family history of tremor was noted. We suspect this patient suffers from neurodegeneration with brain iron accumulation (NBIA).

## Case presentation

**Figure 1:**
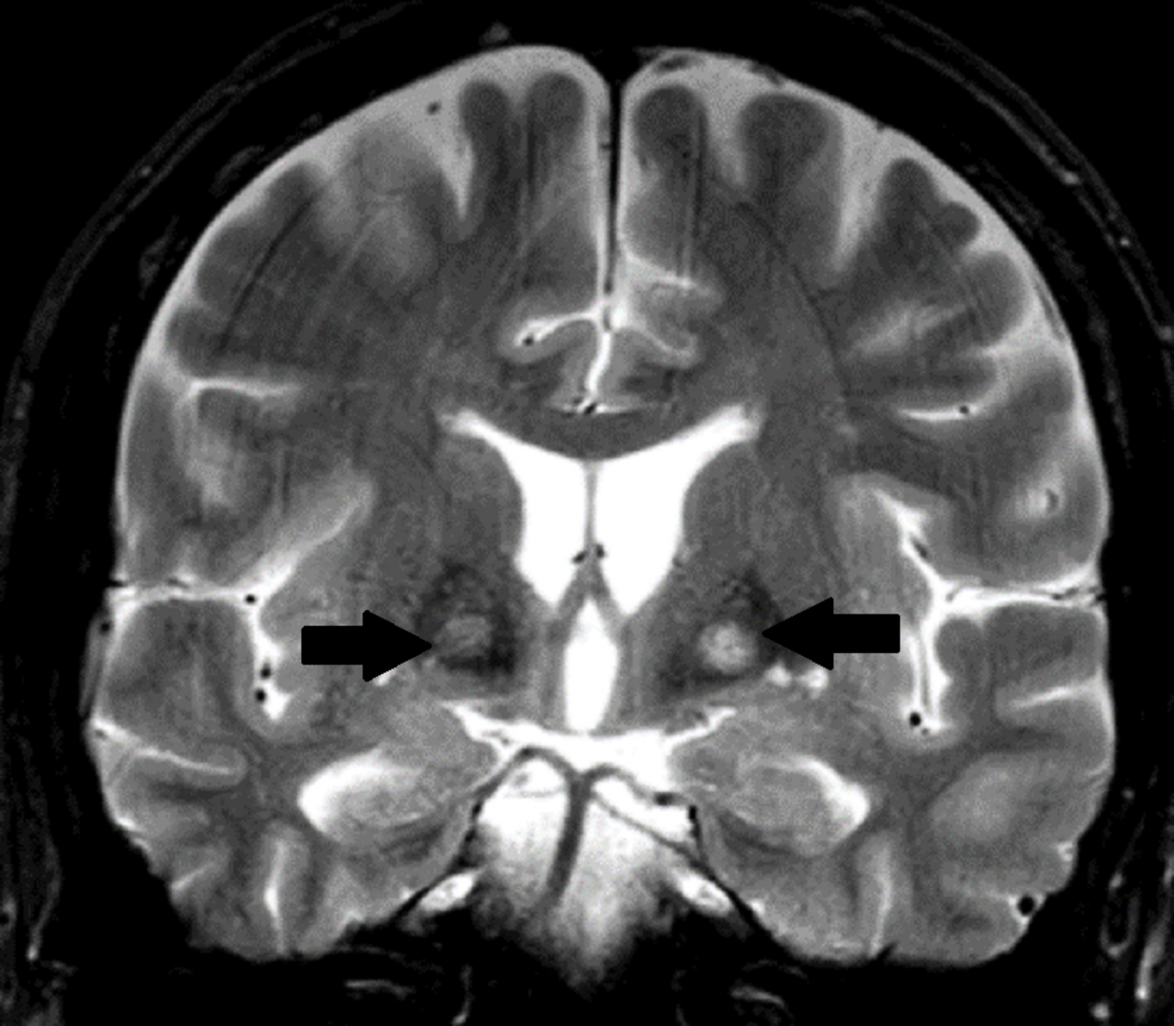
MRI FLAIR Coronal FLAIR sequence on the MRI showed symmetrically decreased T2 signal within the basal ganglia with an area of increased signal intensity (see arrows) suggestive of neutrophil necrosis. FLAIR: fluid-attenuated inversion recovery.

**Figure 2:**
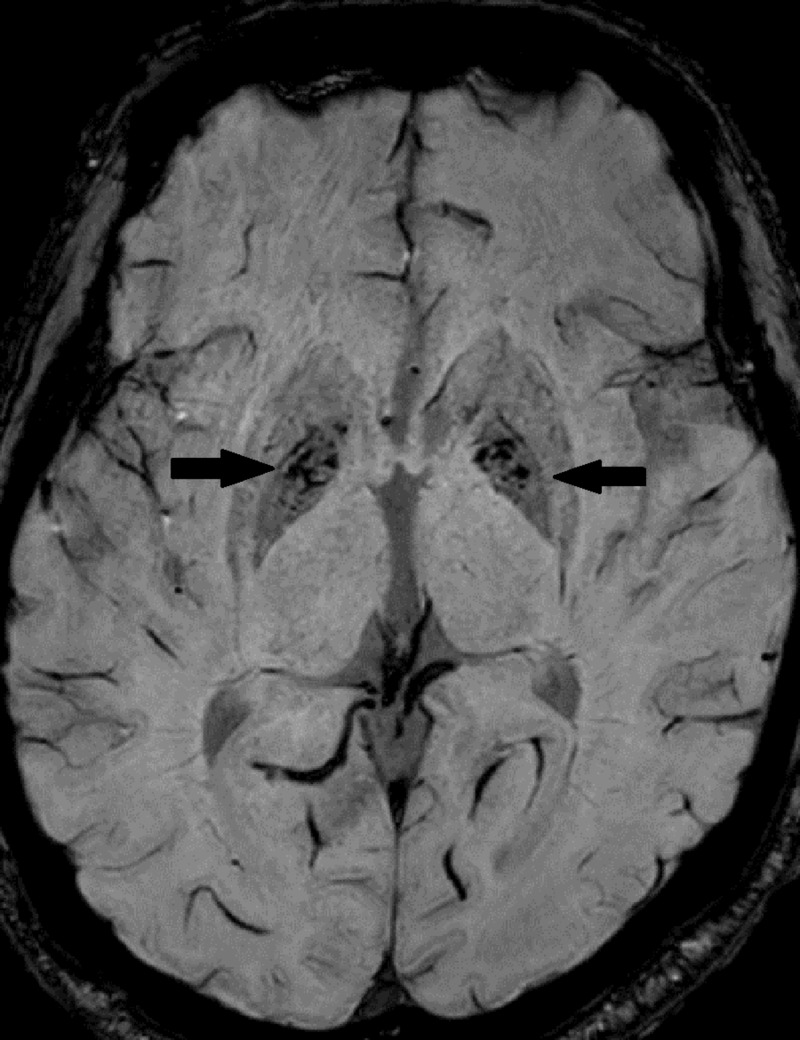
GRE: Transverse Gradient Echo / SWI Susceptibility Weighted Imaging Transverse MRI GRE sequence shows symmetric decreased intensity (dark signal) in the basal ganglia (see arrows). This represents iron accumulation in the basal ganglia. GRE: gradient echo; SWI: susceptibility weighted imaging.

A 68-year-old male patient presented to the neurology clinic with tremor and lightheadedness for several months and a recent history of syncope. Neuroimaging revealed symmetric lesions of the globus pallidus (Figures 1-2) concerning for neurodegeneration with brain iron accumulation (NBIA), formerly known as Hallervorden-Spatz syndrome. MRI revealed increased T2 signal intensity in the medial globus pallidus reminiscent of the eyes of a tiger (the eye-of-the-tiger sign), classically seen due to neutrophil necrosis (Figure 1).

## Discussion

NBIA includes several disorders characterized by MRI changes in the basal ganglia [1]. These disorders are classified based on genetic differences. The most common NBIA disorder is autosomal recessive pantothenate kinase-associated neurodegeneration (PKAN). PKAN is commonly associated with the eye-of-the-tiger sign; for instance, in one study, 100% of the examined PKAN cases had an eye-of-the-tiger sign on brain MRI [2]. A genetic panel for NBIA, specifically PKAN, was ordered for this patient including pantothenate kinase 2 (PanK2) - the genetic marker for pantothenate kinase enzyme. PanK2 is an enzyme needed for coenzyme A production in the mitochondria. Without PanK2, neural tissue degenerates leading to the eye-of-the-tiger sign within the globus pallidus. Unfortunately, the panel was not sent due to insurance issues.

NBIA often presents in children, but it can also present in late adulthood and this might be due to differences in genetic mutations (polymorphisms) [3]. One study found that patients who have mutations which lower the residual activity of the PanK2 enzyme are more likely to develop PKAN between the ages of one and six years [1]. The onset of the disease correlated with the residual activity of the PanK2 enzyme; however, the deterioration of function in patients' daily life did not correlate with any mutations the study examined. The researchers suggest that NBIA may reflect locus heterogeneity where NBIA is caused by mutations in different genes leading to similar phenotypes. It was found, in this study, that the sensitivity of finding a PanK2 enzyme mutation in patients with the eye-of-the-tiger sign on radiology was 68%. This is in contrast to another study that found that 100% of the PKAN cases had an eye-of-the-tiger sign on radiology [2]. It seems that in one study, all of the PKAN cases had an eye-of-the-tiger-sign; however, the eye-of-the-tiger-sign does not guarantee that one will find a PanK2 enzyme mutation on analysis. Other mutations may be involved that lead to an eye-of-the-tiger sign and NBIA.

Clinically, patients have Parkinsonian symptoms including gait abnormalities, tremor, and dementia [4]. Overall, NBIA is a clinical diagnosis that should be pursued if characteristic abnormalities are seen on MRI. NBIA is considered a rare diagnosis with progressive neurodegeneration over time. Ironically, iron chelation therapy has been previously shown to be ineffective; however, a Food and Drug Administration (FDA) approved clinical trial is currently examining the use of the iron chelator, deferiprone, for NBIA [5]. Levodopa and anticholinergic medications may treat Parkinsonian symptoms; baclofen may treat dystonia (uncontrollable muscle contractions), and deep brain stimulation (DBS) may be considered in severe cases of NBIA. 

## Conclusions

This patient has the characteristic MRI finding (eye-of-the-tiger sign) of NBIA, and a history of Parkinsonism (tremor/gait abnormalities) that corroborate this diagnosis. Genetic studies provide further information and sub-type specification.
